# Proteomic Analysis Reveals the Protective Effects of Selenomethionine Against Liver Oxidative Injury in Piglets

**DOI:** 10.3390/ani15131989

**Published:** 2025-07-07

**Authors:** Kai Zhang, Shuhui Yan, Junhong Miao, Wen Li, Zhenxu Li

**Affiliations:** College of Animal Science and Technology, Qingdao Agricultural University, Qingdao 266109, China; 20232209016@stu.qau.edu.cn (S.Y.); junhong-miao@stu.qau.edu.cn (J.M.); 20242109058@stu.qau.edu.cn (W.L.); 20242109019@stu.qau.edu.cn (Z.L.)

**Keywords:** selenomethionine, piglets, liver, oxidative stress, proteomics

## Abstract

This study investigated the protective effects of high selenomethionine (SeMet) supplementation on liver injury caused by oxidative stress in piglets and explored the underlying mechanisms. A total of 18 piglets were randomly assigned to three groups, with six replicates in each group. The control (CON) and diquat (DQ) groups were fed a basal diet supplemented with 0.3 mg Se/kg Se, while the SeMet group received a basal diet supplemented with 1.0 mg Se/kg. The results indicated that SeMet supplementation significantly improved growth performance and increased the serum and liver activities of antioxidant enzymes. Additionally, it reduced the serum and liver levels of malondialdehyde and protein carbonyls in piglets exposed to DQ. Selenoprotein transcriptome analysis showed that the mRNA levels of five selenoprotein genes (*GPX1/3*, *DIO2*, and *SELENOF/M/W*) were significantly upregulated by dietary SeMet supplementation in the liver of DQ-challenged piglets. Proteomic analysis revealed that a total of 3614 proteins were identified in the liver of piglets. Among them, 85 differentially expressed proteins (DEPs) were identified between the CON and DQ groups, 58 DEPs were observed between the DQ and SeMet groups, and 113 DEPs were identified between the CON and SeMet groups. KEGG analysis indicated that most of the DEPs observed among the three groups were involved in fatty acid metabolism, glycolysis/gluconeogenesis, and the PPAR signaling pathway. Together, these results indicate that dietary supplementation with supernutritional SeMet alleviates the negative effects of the DQ challenge on growth performance and liver injury in piglets. This effect is associated with increased antioxidant capacity, enhanced expression of certain selenoprotein genes, and the regulation of fatty acid metabolism.

## Simple Summary

Oxidative stress is recognized as a significant challenge in pig production, particularly during the piglet stage. The liver, as the central organ, is particularly susceptible to oxidative stress. Selenium is an essential trace element for human and animal health, primarily involved in the antioxidant system through selenoproteins. Supernutritional selenium supplementation has been shown to provide various beneficial effects, including antioxidant and anti-stress functions. Therefore, in this study, we aimed to investigate the protective effects and mechanisms of supernutritional selenomethionine (SeMet) supplementation against oxidative liver injury in piglets. Our results indicated that SeMet supplementation improved growth performance and liver health in piglets exposed to oxidative stress. The beneficial effects of SeMet may be correlated with the upregulation of certain selenoprotein mRNA levels (e.g., *GPX1/3*, *DIO2*, and *SELENOF/M/W*). Furthermore, proteomic analysis indicated that the fatty acid metabolism signaling pathway may play a crucial role in the alleviation of liver oxidative damage in piglets by SeMet.

## 1. Introduction

Intensive production has significantly increased pig productivity, but it has also exposed pigs to various stress factors, including weaning stress, environmental factors, feed contamination, and social challenges, which causes reduced growth performance and huge economic losses to the pig industry [1]. These adverse stimuli can disrupt the body’s redox balance, resulting in oxidative stress, particularly in weaning piglets [2,3]. The liver, as the central metabolic organ, is responsible for nutrient utilization and the detoxification of toxins, which is particularly susceptible to oxidative stress [4]. Therefore, reducing liver oxidative stress during the weaning phase is crucial for the health of piglets.

Selenium (Se), an essential trace element, plays a crucial role in mitigating oxidative injury by being incorporated into selenoproteins, such as glutathione peroxidase (GPX) and thioredoxin reductase (TXNRD), which are vital for maintaining cellular redox homeostasis [5,6]. Naturally, Se exists in both inorganic forms (e.g., selenite and selenate) and organic forms (e.g., seleno-amino acids and Se-polysaccharides). Among the various sources of Se, selenomethionine (SeMet) exhibits higher bioavailability and lower toxicity than inorganic Se [7,8,9], making it a promising antioxidant feed additive for alleviating oxidative stress in animals. Considering Se toxicity, the maximum allowable supplementation dose of Se in feed is 0.5 mg/kg, as stipulated by the Code for the Safe Use of Feed Additives in China. Interestingly, recent studies indicate that supernutritional Se supplementation in organic forms, such as SeMet or hydroxy SeMet, provides a protective effect against oxidative stress, heat stress, and lipopolysaccharide challenges in pigs, broilers, and mice [10,11,12,13,14,15,16,17]. However, the potential of supernutritional SeMet supplementation to alleviate oxidative stress in the livers of piglets, as well as the mechanisms underlying the action of SeMet, has not been thoroughly investigated in the literature.

It is widely accepted that Se exerts its biological functions primarily through the synthesis of selenoproteins. Our previous study suggested that supernutritional Se supplementation exerts limited regulatory effects on the liver selenotranscriptome under selenium-adequate conditions [18]. This suggests that the protective effects of supernutritional Se against stress factors that animals may encounter involve mechanisms beyond selenoproteins. Proteomics technology offers valuable insights into alterations in total protein profiles when an organism is subjected to stress factors [19,20]. This approach serves as a promising tool for understanding the nutritional protective mechanisms that combat stress conditions. Our previous research revealed the mechanism by which selenium deficiency induces redox imbalance and inflammation in pig muscle through a proteomics approach [21]. Tang et al. (2022) identified the mechanism by which selenium deficiency induces pathological cardiac lipid metabolic remodeling in pigs using proteomics technology [22].

Diquat is a bipyridyl herbicide that utilizes molecular oxygen to generate superoxide anion radicals, which are frequently employed as inducers of oxidative stress. Therefore, in the present study, we evaluated the protective effects of dietary supernutritional SeMet supplementation on growth performance, liver morphology, and selenoprotein expression in oxidatively stressed piglets induced by diquat. Furthermore, we examined the changes in the liver proteomics of piglets to elucidate the mechanisms by which supernutritional SeMet supplementation alleviates liver oxidative stress.

## 2. Materials and Methods

### 2.1. Chemicals and Reagents

SeMet was purchased from J&K Scientific Co., Ltd. (Beijing, China). Diquat dibromide monohydrate (DQ) was obtained from Toronto Research Chemicals (Toronto, ON, Canada).

### 2.2. Animals, Diets, and Sample Collection

A total of 18 male piglets (Duroc × Landrace × Large Yorkshire, 28 days old) with an average body weight of 8.11 ± 0.24 kg were randomly assigned to three groups with six replicates in each group. The control (Con) group and DQ group were fed a basal diet (Supplementary Table S1, containing 0.3 mg Se/kg), as recommended by the China Nutrition Requirement of Swine (GB/T 39235-2020 [23]), while the SeMet group was fed a basal diet supplemented with SeMet (1.0 mg Se/kg diet), according to a previous report [8,10]. SeMet was added to the premix feed, replacing some carriers. At 25 days, the DQ and SeMet groups were administered with DQ (10 mg/kg body weight, in sterile saline) via intraperitoneal injection to induce oxidative stress in piglets, while the Con groups received equal volumes of saline. The dose of DQ challenged with piglets was according to a previous report [24]. Piglets provided free access to water and feed. The deworming and vaccination history is as follows: mycoplasma hyopneumoniae was administered at 7 days old, porcine circovirus was given at 14 days old, and classical swine fever virus was administered at 35 days old. The experiment lasted for 28 days. The initial (day 1) and final (day 28) body weights were measured to calculate the average daily gain. At the end of the experiment, all piglets were euthanized after fasting for 12 h, and liver samples were immediately collected, fixed in 4% paraformaldehyde for histological analysis. Some portions were frozen in liquid nitrogen and then stored at −80 °C.

### 2.3. Diet, Serum, and Liver Selenium Content Analysis

A quantity of 0.5 g or mL of samples was mixed with 8 milliliters of nitric acid. The mixture was allowed to sit at room temperature overnight. Subsequently, it was transferred to a microwave digestion system (CEM-MARSX^®^, CEM Corporation, Matthews, NC, USA), where it underwent a sequential digestion process at 120 °C, 150 °C, and 190 °C, in accordance with the established protocol [7]. The determination of selenium was performed using an Agilent 7900 Inductively Coupled Plasma Mass Spectrometer (ICP-MS) (Agilent Technologies, Santa Clara, CA, USA), as detailed in our previous report [7].

### 2.4. Histological Analysis

The fixed liver tissues were embedded in paraffin and cut into 5 μm sections for hematoxylin-eosin staining according to the previous report [21]. Subsequently, the histological characteristics of liver tissues were observed using an Olympus microscope (Olympus, Tokyo, Japan).

### 2.5. Antioxidant Capacity Analysis

The enzymatic activities of superoxide dismutase (SOD), catalase (CAT), malondialdehyde (MDA), and glutathione peroxidase (GPX) were quantitatively analyzed using commercially available assay kits (Nanjing Jiancheng Bioengineering Institute, Nanjing, Jiangsu Province, China). Concurrently, immunological parameters including interleukin-1β (IL-1β), interleukin-4 (IL-4), interleukin-6 (IL-6), interleukin-10 (IL-10), immunoglobulin A (IgA), and immunoglobulin M (IgM) were measured by enzyme-linked immunosorbent assay (ELISA) kits (Shanghai Enzyme-linked Biotechnology Co., Ltd., Shanghai, China). Serum samples and supernatants from liver tissue homogenates were analyzed for these parameters. Absorbance readings were acquired using a Multiskan Spectrum microplate spectrophotometer (SpectraMax iD3; Molecular Devices, Shanghai, China), with all experimental procedures strictly adhering to manufacturers’ protocols.

### 2.6. Determination of mRNA Expression Levels by RT-qPCR

The protocols for total RNA isolation, RNA purity and integrity assessment, and cDNA synthesis are detailed in our previous report [7]. The abundance of selenoprotein mRNA was determined using a Bio-Rad CFX96™ Real-Time PCR system (Bio-Rad Laboratories, Hercules, CA, USA) and quantified using the 2^−ΔΔCt^ method. The primers were reported in our previous studies [7,18,21].

### 2.7. Proteomic Analysis

Four liver samples from each of the CON, DQ, and SeMet groups were subjected to quantitative proteomic analysis. Protein extraction, trypsin digestion, and desalting protocols were performed in accordance with our previous description [21]. The digested peptides were conducted on an EASY-nLC 1200 system (Thermo Fisher, Waltham, MA, USA) and separated on a C18 column (75 μm × 250 mm, IonOpticks, New York, NY, USA) via linear gradient elution. The mobile phases consisted of water with 0.1% formic acid and 2% aqueous (A) and 80% aqueous acetonitrile with 0.1% formic acid (B), with a flow rate of 250 nL/min. The gradient was as follows: 0–45 min, 3–28% B; 45–50 min, 28–44% B; 50–55 min, 44–90% B, maintained until 60 min.

The eluate was passed into the tims TOF Pro 2 MS (Bruker, Karlsruhe, Germany) for 4D proteomics analysis. The analysis was conducted in the DIA-PASEF mode with positive ion detection. The ion source voltage was set to 1.5 kV, and both MS and MS/MS spectra were acquired using a TOF mass analyzer (Bruker, Karlsruhe, Germany). The accumulation and ramp times were configured at 100 ms each, with an MS scan range of *m*/*z* 100–1700. The ion mobility coefficient was maintained within 0.6–1.6 V·s/cm^2^. A total of 64 DIA-PASEF windows (25 isolation windows) were applied, with each TIMS cycle consisting of 1 MS scan and 10 PASEF MS/MS scans during data acquisition. Precursors with charge states 0–5 were selected for MS/MS analysis, employing a dynamic exclusion duration of 24 s to minimize redundant precursor selection. Higher-energy collisional dissociation was utilized for fragmentation. DIA raw data were analyzed using the Spectronaut™ platform (version 16.0) with the Sus scrofa (pig) UniProt reference proteome database. Retention time alignment was conducted using iRT calibration. The quantification parameters included six unique peptides per protein, with three fragment ions per peptide; a 1% false discovery rate (FDR) threshold at both the protein and peptide levels; a 99% peptide confidence cutoff; and an extracted ion chromatogram (XIC) mass tolerance of ±75 ppm. Shared peptides and post-translationally modified variants were excluded from quantification. Relative protein abundance was determined by summing the normalized peak areas from high-confidence transitions.

### 2.8. Validation of Proteomics Results Using Commercial Assay Kits

To further validate the proteomics results, three DEPs (FASN, LDHB, and G6PD) were selected to determine their activity using commercial assay kits (Biosharp, Hefei, China), following the manufacturers’ protocols.

### 2.9. Statistical Analysis

Data are presented as the mean ± SEM. The significant differences in growth performance, redox parameters, and mRNA levels were assessed using ANOVA with SPSS 26.0 software, followed by Duncan’s multiple comparison tests with a significance level set at *p* < 0.05. For proteomics analysis, differentially expressed proteins (DEPs) were selected based on the criteria of *p* < 0.05 and a fold change (FC) ≥ 1.5 or ≤0.66. Volcano plot, Venn diagram, and heatmap analyses were conducted using an online platform (https://www.bioinformatics.com.cn/, accessed on 16 May 2025). Gene Ontology (GO) enrichment and Kyoto Encyclopedia of Genes and Genomes (KEGG) pathway analyses were performed using DAVID (https://davidbioinformatics.nih.gov/, accessed on 16 May 2025) and Metascape (https://metascape.org/, accessed on 16 May 2025).

## 3. Results

### 3.1. Growth Performance and Liver Morphology

**Figure 1:**
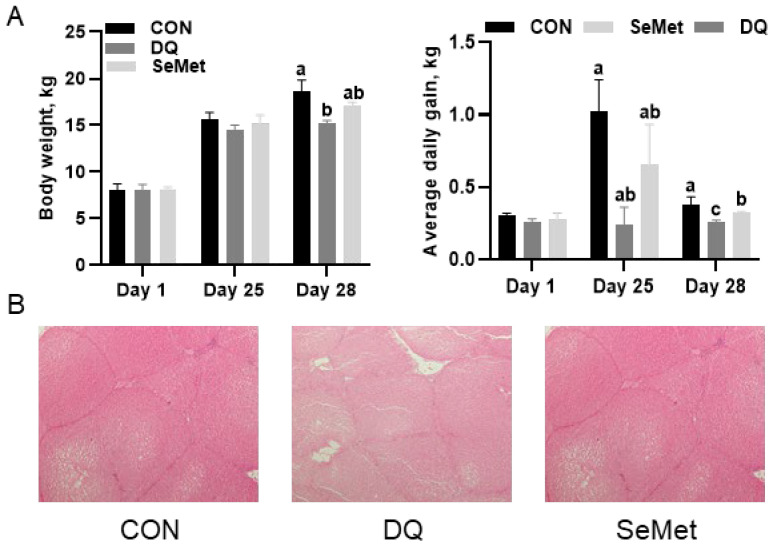
Effects of selenomethionine supplementation on growth performance (**A**) and liver morphology (**B**) of piglets exposed to diquat and selenomethionine. CON, control received a basal diet; DQ, control + diquat exposure; SeMet, control + selenomethionine supplementation + diquat exposure. Different letters mean significantly different for different groups at *p* < 0.05.

The effects of SeMet on growth performance, Se status, and liver morphology in oxidative piglets are illustrated in Figure 1. No significant effects of SeMet on body weight and average daily gain (ADG) were observed among the CON, DQ, and SeMet groups from days 1 to 25 (*p* > 0.05). The final body weight in the DQ group (15.20 ± 0.32 kg) was significantly lower (*p* < 0.05) than that in the CON (18.68 ± 1.16 kg) and SeMet (17.10 ± 0.39 kg) groups. During days 25 to 28, the ADG in the CON group (1.02 ± 0.22 kg) was significantly higher (*p* < 0.05) than that in the DQ group (0.24 ± 0.12 kg). Dietary supplementation with SeMet increased (*p* < 0.05) the ADG in the DQ group by 170%. Over the entire period from days 1 to 28, the ADG in the DQ group (0.26 ± 0.01 kg) was lower (*p* < 0.05) than that in both the CON group (0.38 ± 0.02 kg) and the SeMet group (0.32 ± 0.01 kg). Dietary supplementation with SeMet effectively improved the ADG of piglets challenged with DQ by 23%; however, these values remained significantly lower (*p* < 0.05) than those observed in the CON group. In comparison to the CON group, DQ-treated piglets exhibited significant hepatic injury. The SeMet group demonstrated a marked reduction in DQ-induced hepatic injury.

### 3.2. Serum and Liver Antioxidant Capacity

**Figure 2:**
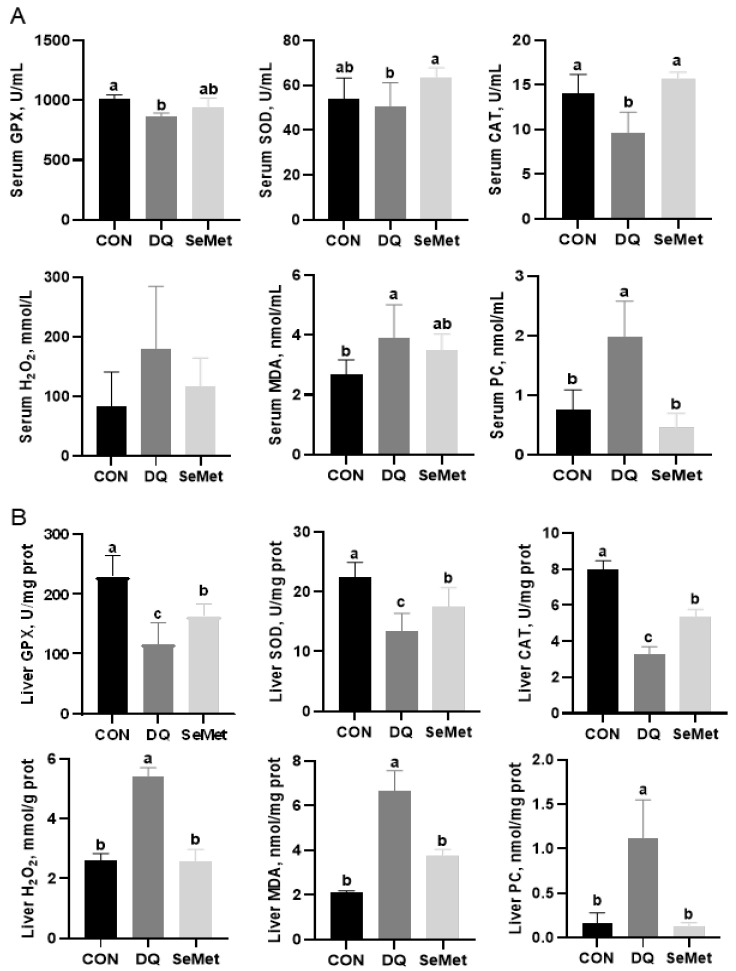
Effects of selenomethionine supplementation on serum (**A**) and liver (**B**) antioxidant capacity in piglets exposed to diquat and selenomethionine. CON, control received a basal diet; DQ, control + diquat exposure; SeMet, control + selenomethionine supplementation + diquat exposure. Different letters mean significantly different for different groups at *p* < 0.05.

As illustrated in Figure 2A, DQ-challenged piglets exhibited significantly decreased serum GPX (−14%) and CAT (−32%) activities compared to their CON counterparts (*p* < 0.05). This reduction was accompanied by increased levels of MDA (+45%) and PC (+159%) (*p* < 0.05). Supplementation with SeMet effectively restored the antioxidant capacity impaired by DQ, elevating serum GPX (+9%) and SOD (+25%) activities to levels comparable to those of the CON group. Figure 2B illustrates the hepatoprotective effects of SeMet against oxidative injury. The activities of hepatic GPX (−49%), SOD (−40%), and CAT (−59%) in DQ-treated piglets were significantly reduced compared to the CON group (*p* < 0.05). In contrast, the concentrations of hepatic H_2_O_2_, MDA, and PC showed increases ranging from 1- to 6-fold (*p* < 0.05). SeMet supplementation significantly increased hepatic antioxidant enzyme activities (GPX: +40%, SOD: +30%, CAT: +63% compared to the DQ group) while simultaneously reducing markers of oxidative stress (H_2_O_2_: −53%, MDA: −44%, PC: −88% compared to the DQ group). Notably, despite these enhancements, hepatic antioxidant activities in the SeMet group remained 22–33% lower than those in the control group (*p* < 0.05).

### 3.3. Selenium Status and Liver Selenoprotein Transcriptome

**Figure 3:**
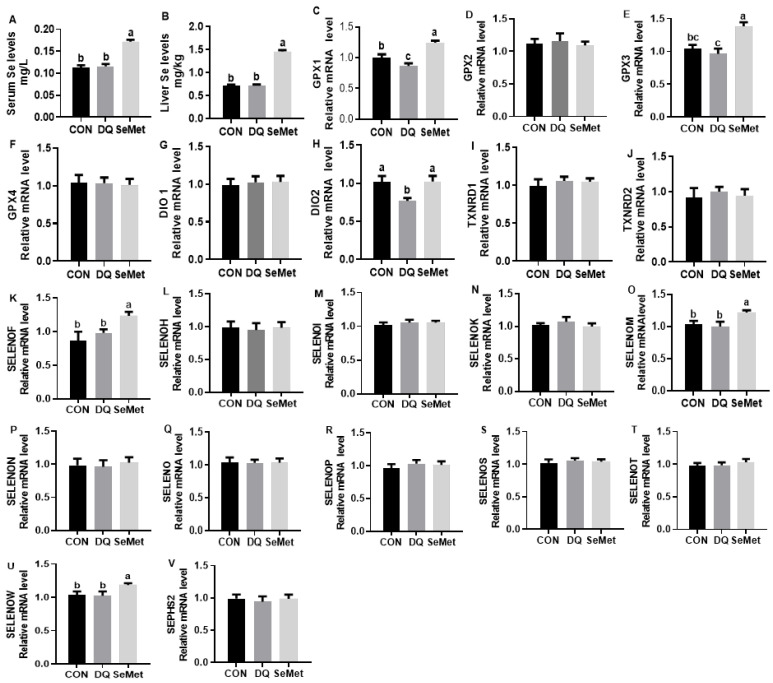
Effects of selenomethionine supplementation on selenium status (**A**,**B**) and liver selenoprotein mRNA levels (**C**–**V**) in piglets exposed to diquat and selenomethionine. CON, control received a basal diet; DQ, control + diquat exposure; SeMet, control + selenomethionine supplementation + diquat exposure. Different letters mean significantly different for different groups at *p* < 0.05.

The DQ challenge has no significant effects on the serum and liver Se levels in piglets (Figure 3A,B). In contrast, dietary supplementation with SeMet significantly increased the Se content in both the serum and liver tissues of piglets compared to the CON and DQ groups (*p* < 0.05). As shown in Figure 3, the DQ challenge had a limited effect on the mRNA expression of selenoprotein genes, with the exception of a downregulation in the expression of *GPX1* and *DIO2* (*p* < 0.05). In contrast, dietary supplementation with SeMet significantly upregulated the expression of *GPX1*, *GPX3*, *DIO2*, *SELENOF*, *SELENOM*, and *SELENOW* in DQ-challenged piglets (*p* < 0.05).

### 3.4. Protein Identification and DEPs Analysis

**Figure 4:**
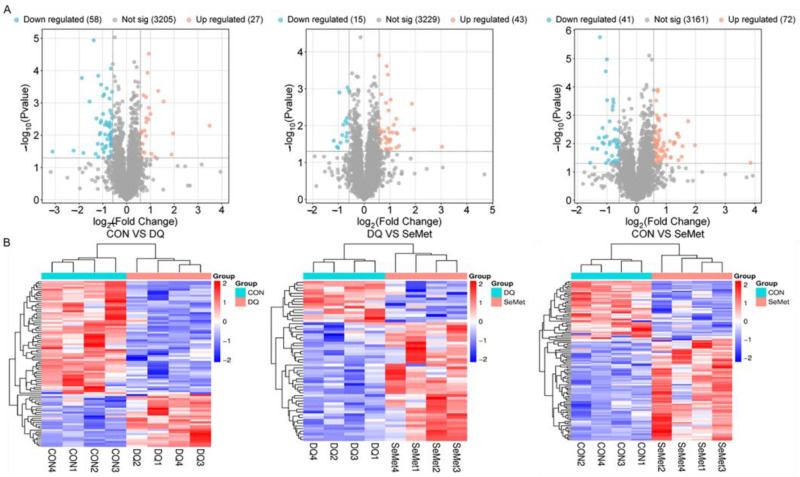
Volcano plot (**A**) and heatmap (**B**) analysis of liver proteome profiles in piglets. CON, control received a basal diet; DQ, control + diquat exposure; SeMet, control + selenomethionine supplementation + diquat exposure.

A total of 3614 proteins were identified in the livers of piglets from the CON, DQ, and SeMet groups using 4D label-free proteomics (Supplementary Table S2). The lists of DEPs between the CON and DQ groups, the DQ and SeMet groups, and the CON and SeMet groups are presented in Supplementary Tables S3, S4, and S5, respectively. The volcano plot (Figure 4A) and heatmap (Figure 4B) analyses indicate that 85 DEPs were identified between the CON and DQ groups, of which 27 DEPs were upregulated and 58 DEPs were downregulated in the DQ group compared to the CON group. Fifty-eight DEPs were observed between the DQ and SeMet groups, with 43 DEPs upregulated in the SeMet group compared to the DQ group. Additionally, 113 DEPs (72 upregulated and 41 downregulated) were observed in the SeMet group compared to the CON group.

**Figure 5:**
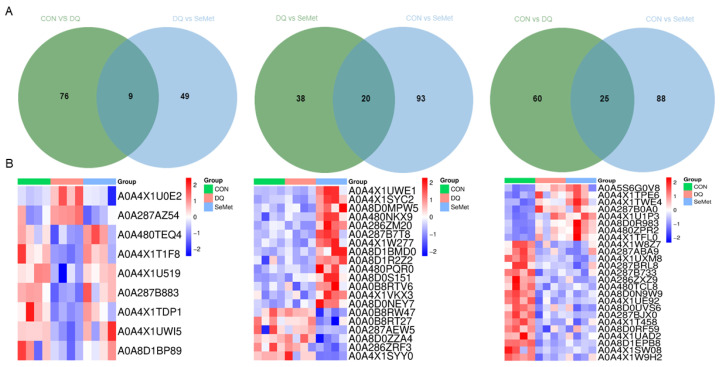
Venn diagram (**A**) and heatmap (**B**) analysis of the common differentially expressed proteins in the liver among the CON, DQ, and SeMet groups. CON, control received a basal diet; DQ, control + diquat exposure; SeMet, control + selenomethionine supplementation + diquat exposure.

The Venn diagram analysis was employed to identify the common DEPs among the CON, DQ, and SeMet groups (Figure 5A). Compared to the DQ groups, nine DEPs (Table 1 and Figure 5B) were identified in both the CON and SeMet groups. Among these, two DEPs (A0A4XIU0E2 and A0A287AZ54) exhibited significant increases following DQ treatment, while seven DEPs were decreased in the DQ group. Notably, all nine DEPs in the SeMet group were restored to levels comparable to those of the CON group. Twenty DEPs were observed in the SeMet group compared to the CON and DQ groups, with 16 DEPs upregulated and 4 DEPs downregulated. In the CON group, 25 DEPs were identified compared to the DQ and SeMet groups, consisting of 8 DEPs downregulated and 17 DEPs upregulated.

### 3.5. Functional Enrichment Analysis of DEPs

**Figure 6:**
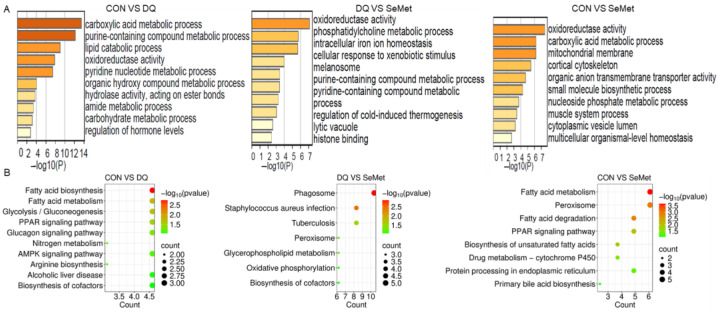
Go (**A**) and KEGG (**B**) enrichment analysis of the differentially expressed proteins identified among the CON, DQ, and SeMet groups. CON, control received a basal diet; DQ, control + diquat exposure; SeMet, control + selenomethionine supplementation + diquat exposure.

GO and KEGG enrichment analysis (Figure 6A,B) were conducted to explore the biological functions of the DEPs identified among the CON, DQ, and SeMet groups. According to the GO enrichment analysis, the DEPs identified between the CON and DQ groups were primarily involved in the carboxylic acid metabolic process, purine-containing compound metabolic process, lipid catabolic process, oxidoreductase activity, and hydrolase activity. The DEPs among the DQ and SeMet groups were mainly involved in oxidoreductase activity, the phosphatidylcholine metabolic process, intracellular iron ion homeostasis, cellular response to xenobiotic stimulus, and melanosome. The DEPs between the CON and SeMet groups were primarily involved in oxidoreductase activity, the carboxylic acid metabolic process, mitochondrial membrane, cortical cytoskeleton, and organic anion transmembrane transporter activity. KEGG analysis showed that the DEPs observed among the CON, DQ, and SeMet groups were primarily involved in fatty acid biosynthesis, the PPAR signaling pathway, phagosome, peroxisome, glycolysis/gluconeogenesis, and glycerophospholipid metabolism.

### 3.6. Validation of Proteomics Results

**Figure 7:**
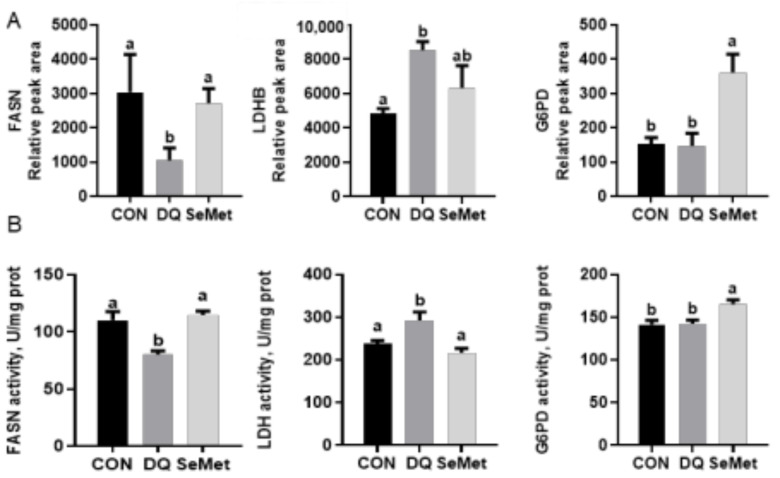
Validation of liver proteome results (**A**) using the ELISA (**B**) approach. CON, control received a basal diet; DQ, control + diquat exposure; SeMet, control + selenomethionine supplementation + diquat exposure. Different letters mean significantly different for different groups at *p* < 0.05.

As shown in Figure 7, the activities of three DEPs (FASN, LDHB, and G6PD) were determined using commercial assay kits. Compared to the CON group, the DQ challenge significantly decreased the activity of FASN and increased the activity of LDH (*p* < 0.05). In contrast, dietary supplementation with SeMet significantly increased the activities of FASN and G6PD, while decreasing the activity of LDH in the liver of DQ-challenged piglets (*p* < 0.05) (Figure 7B). These results were consistent with those observed in the proteomics analysis (Figure 7A), further supporting the validity of the proteomic approach.

## 4. Discussion

Weaned piglets exhibit increased susceptibility to oxidative stress compared to other developmental stages, resulting in impaired growth performance and higher mortality rates. This presents a significant challenge to the pig production industry. DQ is a moderately toxic chemical that generates superoxide anion radicals and H_2_O_2_, which can disrupt the redox balance and lead to oxidative stress [25]. Therefore, in this study, DQ exposure was utilized to establish an oxidative stress model for piglets, aiming to investigate the protective effects and mechanism of supernutritional SeMet supplementation on weaning piglets’ exposure to oxidative stress. The negative impact of oxidative stress induced by DQ on growth performance in piglets has been well documented in previous research [24,26,27]. In the present study, the DQ challenge exhibited a sharp decrease in the ADG and final body weight of piglets, which is consistent with previous findings. The liver is the primary target organ of DQ. Redox imbalance is regarded as the primary mechanism underlying liver injury induced by DQ challenge. In mammals, GPX, SOD, and CAT are the primary antioxidant enzymes that convert superoxide radicals into H_2_O_2_ and subsequently into H_2_O [28]. MDA and PC are recognized as biomarkers for lipid and protein peroxides, with their levels serving as indirect indicators of the extent of oxidative damage [29,30]. In the present study, the DQ challenge resulted in significant liver injury and decreased the activities of GPX, SOD, and CAT, while increasing the levels of MDA and PC in the serum and liver of piglets, which was consistent with previous findings reported in piglets [24,26,31]. These results indicate that the DQ challenge causes oxidative stress and liver injury, which supports the successful establishment of the oxidative stress model in piglets. Previous studies have confirmed the beneficial effects of organic Se (e.g., SeMet and hydroxyl-SeMet) on improving growth performance, enhancing antioxidant capacity, and alleviating liver, intestinal, and skeletal muscle injuries caused by oxidative stress and heat stress in pigs and broilers [12,14,15,32,33,34]. Similarly, in this study, dietary supplementation with SeMet enhanced growth performance, improved antioxidant capacity, and mitigated liver injury in DQ-challenged piglets. These results confirm the beneficial effects of supernutritional SeMet supplementation on the growth performance and liver health of piglets exposed to oxidative stress.

Se exerts most of its known biological functions through selenoproteins [35]. To investigate how SeMet supplementation enhances growth performance and mitigates liver injury in piglets, we assessed the mRNA expression of the liver selenotranscriptome in response to DQ exposure and SeMet supplementation. The results indicated that, with the exception of *GPX1* and *DIO2*, the DQ challenge had limited effects on the mRNA expression of the liver selenotranscriptome, consistent with a previous report [10]. Furthermore, SeMet supplementation upregulated six selenoprotein mRNA expressions (*GPX1*, *GPX3*, *DIO2*, *SELENOF*, *SELENOM*, and *SELENOW*) in the liver of DQ-exposed piglets. Jing et al. (2022) reported that dietary hydroxy-SeMet supplementation at a dosage of 0.9 mg/kg increased the expression of 22 selenoprotein genes in the livers of oxidative-stressed finishing pigs [10]. Unlike our recent findings, the CON and oxidative stress groups in the aforementioned study were given Se-deficient diets, and the pigs were in the finishing stage. A previous report has confirmed the age-dependent changes in the expression of selenoproteins in the liver of pigs [36]. This may clarify the observed differences in selenoprotein expression. Notably, most of the altered selenoproteins are involved in regulating redox status (e.g., *GPX1*, *GPX3*, and *SELENOW*) or are located in the endoplasmic reticulum (e.g., *DIO2*, *SELENOF*, and *SELENOM*) [37,38]. GPX1, GPX3, and SELENOW are the most important selenoproteins involved in redox regulation [37,39]. DIO2 plays a crucial role in regulating thyroid hormone activity. SELENOF, a selenoprotein functionally linked to protein folding, is regulated by selenium status and various stress factors [40]. SELENOM is a selenoprotein with redox activity that enhances antioxidant capacity and alleviates endoplasmic reticulum stress [41]. The upregulation of endoplasmic reticulum-resident selenoproteins indicates that SeMet may alleviate liver injury by regulating endoplasmic reticulum homeostasis in DQ-challenged piglets.

Furthermore, a 4D proteomics analysis was conducted to characterize the alterations in proteomic profiles in response to the DQ challenge and SeMet supplementation. The levels of fructose-1,6-bisphosphatase 1, lactate dehydrogenase, cytochrome P450 3A, and UDP-glucuronosyltransferases were elevated, while serine/threonine-protein phosphatase, purine nucleoside phosphorylase, superoxide dismutase, and fatty acid synthase were decreased following DQ exposure in the liver of pigs. Hu et al. (2021) reported that fructose-1,6-bisphosphatase 1 may exacerbate oxidative stress-induced apoptosis by inhibiting the Nrf2 pathway [42]. The elevated levels of lactate dehydrogenase in serum are considered a biomarker for liver injury [43]. UDP-glucuronosyltransferases and cytochrome P450 are essential phase II drug-metabolizing enzymes that catalyze the glucuronidation reaction, facilitating the excretion of drugs and various endogenous substances [44]. The upregulation of UDP glucuronosyltransferases and cytochrome P450 in the liver of piglets may be attributed to a compensatory reaction induced by DQ exposure. It is important to note that dietary supplementation with SeMet significantly reversed the aforementioned trends in the liver of the DQ group. Previous report findings suggested that oxidative stress causes disorders in pig liver lipid metabolism. However, hydroxyl-SeMet restored the activity of liver lipid metabolism enzymes and increased the expression of synthesis-related genes [10]. According to the KEGG enrichment analysis, most of the DEPs observed in the liver of the CON, DQ, and SeMet groups were primarily involved in fatty acid metabolism, glycolysis/gluconeogenesis, and the PPAR signaling pathway. Consistent with previous research, the levels and activity of fatty acid synthase were significantly upregulated by SeMet supplementation. Zhao et al. (2016) reported that a high Se (3.0 mg Se/kg, provided as Se-enriched yeast) diet upregulated the mRNA levels and enzyme activity of liver fatty acid synthase-related genes compared to a diet containing 0.3 mg Se/kg in pigs [45]. Taken together, the protective effects of SeMet in alleviating liver injury may occur through the regulation of the fatty acid metabolism pathway. The endoplasmic reticulum is a major site for coordinating lipid synthesis, transport, and metabolism. Previous studies suggested that endoplasmic reticulum-resident selenoproteins (e.g., SELENOF, SELENOM, DIO2) were involved in lipid metabolism [41,46,47]. Therefore, the regulation of SeMet on liver lipid metabolism in DQ-challenged piglets may occur through endoplasmic reticulum-resident selenoproteins, including *SELENOF*, *SELENOM*, and *DIO2*.

## 5. Conclusions

Our results confirmed the protective effects of SeMet supplementation on the growth performance, antioxidant activity (9–63%), and liver health of piglets exposed to DQ. Selenoprotein transcriptome and proteomics analysis revealed that these beneficial effects are achieved by regulating fatty acid metabolism, glycolysis/gluconeogenesis, and the PPAR signaling pathway. In particular, the antioxidant and endoplasmic reticulum-resident selenoprotein genes (*GPX1*, *GPX3*, *DIO2*, *SELENOF*, *SELENOM*, and *SELENOW*) are influenced following supernutritional SeMet supplementation.

## Figures and Tables

**Table 1:** The list of nine common DEPs between the CON and DQ groups, as well as between the DQ and SeMet groups.

Accession	Gene Symbol	Protein Name	Molecular Function	Biological Process
A0A4X1U0E2	QPRT	nicotinate-nucleotide pyrophosphorylase	nicotinate-nucleotide diphosphorylase (carboxylating) activity	NAD biosynthetic process
A0A287AZ54	PDXP	pyridoxal phosphatase	pyridoxal phosphatase activity	pyridoxal phosphate catabolic process
A0A480TEQ4		apolipoprotein A2	lipid binding	lipid transport
A0A4X1T1F8		apolipoprotein A1		
A0A4X1U519		C1q domain-containing protein	-	-
A0A287B883	PTMS	parathymosin	histone binding	negative regulation of apoptotic process
A0A4X1TDP1	PTMSA	prothymosin alpha	-	-
A0A4X1UWI5	ETNPPL	ethanolamine-phosphate phospho-lyase	transaminase activity	-
A0A8D1BP89	FASN	fatty acid synthase	hydrolase	fatty acid biosynthesis

- Not annotation in UniProt. CON, control received a basal diet; DQ, control + diquat exposure; SeMet, control + selenomethionine supplementation + diquat exposure.

## Data Availability

All data sets collected and analyzed in this study are available at the request of the corresponding author.

## Supplementary Materials

The following supporting information can be downloaded at: https://www.mdpi.com/article/10.3390/ani15131989/s1, Table S1: Composition and nutrient levels of the basal diet; Table S2: The list of detailed information on proteins identified in the livers of piglets; Table S3: The list of DEPs between the CON and DQ groups; Table S4: The list of DEPs between the DQ and SeMet groups; Table S5: The list of DEPs between the CON and SeMet groups.
