# Supplementary material for: Proteomic Analysis Reveals the Protective Effects of Selenomethionine Against Liver Oxidative Injury in Piglets
Source: Animals (Basel). 2025 Jul 7;15(13):1989. doi: 10.3390/ani15131989 (PMC12248808; doi:10.3390/ani15131989)
Supplement: Supplementary file 1 [file animals-15-01989-s001.zip › Supplementary Table S3, S4, and S5.pdf]

Table S3 The list of DEPs between the CON and DQ groups

Accession	Gene symbol	Protein_name	P value	Log2FC
A0A4X1SQ08	LOC110260333	Hydroxymethylglutaryl-CoA synthase	0.0051	3.49
A0A8D1I2A8	AGXT	Alanine--glyoxylate aminotransferase	0.0089	1.96
A0A480YSK3		Glycine N-methyltransferase	0.0398	1.89
A0A8D0PT98		Alanine--glyoxylate aminotransferase	0.0009	1.56
A0A5S6G0V8	CA3	Carbonic anhydrase	0.0004	1.33
A0A4X1TPE6		Cytochrome c oxidase subunit NDUFA4	0.0469	1.19
A0A4X1THU4	TOR1A	Torsin	0.0376	1.04
A0A287A9G4	ASS1	Argininosuccinate synthase	0.0022	1.00
A0A4X1SKS3	HAL	Histidine ammonia-lyase	0.0014	0.94
Q95JC8	ARG1	Arginase-1	0.0000	0.93
A0A4X1U0E2	QPRT	Nicotinate-nucleotide pyrophosphorylase [carboxylating]	0.0032	0.92
P00636	FBP1	Fructose-1,6-bisphosphatase 1	0.0001	0.88
A0A4X1TWE4	RCN2	Reticulocalbin-2	0.0039	0.83
A0A287B0A0	BCO2	Carotenoid-cleaving dioxygenase	0.0030	0.83
A0A4X1VAI4	LDHB	L-lactate dehydrogenase	0.0007	0.82
A0A288CFV1	UBE2D3	Ubiquitin conjugating enzyme E2 D3	0.0106	0.81
A0A286ZXK8	MCAT	Malonyl-CoA-acyl carrier protein transacylase	0.0072	0.76
A0A8D1KR24	CYP3A46	Cytochrome P450 3A	0.0345	0.75
A0A4X1W0G3	BCAT2		0.0006	0.71
A0A287AZ54	PDXP	Pyridoxal phosphatase	0.0186	0.67
A0A4X1U1P3	HSD17B13	17-beta-hydroxysteroid dehydrogenase 13 isoform A	0.0327	0.66
A0A8D0LR60		Glycine N-acyltransferase-like protein	0.0489	0.64
A0A8D0R983	CBR4	3-ketoacyl-reductase beta subunit	0.0075	0.62
A0A480ZPR2		Estradiol 17-beta-dehydrogenase 8	0.0084	0.62
A0A287B4Z6	SARDH	Sarcosine dehydrogenase	0.0476	0.62
A0A8D1BHM4		Fructose-bisphosphate aldolase	0.0180	0.60
A0A4X1TFL0	UGT1A6	UDP-glucuronosyltransferase	0.0087	0.60
A0A480F6B5		Outer mitochondrial transmembrane helix translocase	0.0077	-0.59
A0A286ZN79	TOM1L1	Target of myb1 like 1 membrane trafficking protein	0.0197	-0.61
A0A4X1VRE9	CD5L	Scavenger receptor cysteine-rich type 1 protein M130-like	0.0030	-0.61
A0A4X1UM02	PDLIM1	PDZ and LIM domain protein 1	0.0005	-0.62
A0A8D1NT38		Acyl-coenzyme A diphosphatase NUDT19	0.0450	-0.65
A0A0B8RZN7	CIAPIN1	Anamorsin	0.0021	-0.65
A0A287A372	SORBS2	Sorbin and SH3 domain containing 2	0.0047	-0.65

A0A287AX01	MAPK14	Mitogen-activated protein kinase	0.0221	-0.66
A0A286ZVG1	PGAM5	Serine/threonine-protein phosphatase	0.0128	-0.66
A0A4X1UYU0		Myotubularin related protein 14	0.0425	-0.66
A0A287A2Y1	PNP	Purine nucleoside phosphorylase	0.0081	-0.66
A0A4X1W8Z7		Alpha-1B-glycoprotein	0.0148	-0.66
A0A287ABA9	LOC110257570	TRPM8 channel-associated factor 2 isoform X1	0.0134	-0.66
A0A4X1UXM8	CYGB	superoxide dismutase	0.0001	-0.66
A0A075B7H9		Ig-like domain-containing protein	0.0180	-0.66
A0A480SUZ7		Fetuin-B isoform 1	0.0122	-0.66
A0A480TEQ4		Apolipoprotein A-II	0.0198	-0.66
A0A287BRL8	MARCKS	Myristoylated alanine-rich C-kinase substrate	0.0324	-0.67
A0A4X1UIB6	LOC110259958	Hemoglobin subunit alpha	0.0100	-0.68
A0A287B733	FBXL2	F-box and leucine rich repeat protein 2	0.0017	-0.69
A0A286ZXZ9	EPB41L2	Erythrocyte membrane protein band 4.1 like 2	0.0057	-0.70
A0A287BJ64	AZGP1	Alpha-2-glycoprotein 1	0.0095	-0.70
A0A4X1T1F8		Apolipoprotein A-I	0.0040	-0.71
A0A287BQR3	FCN2	Ficolin 2	0.0007	-0.72
A0A8D1GGQ5		IF rod domain-containing protein	0.0478	-0.72
A0A481B9A6	HRG	Histidine-rich glycoprotein	0.0102	-0.74
A0A480TCL8		Alpha-1-acid glycoprotein	0.0051	-0.78
A0A4X1TK83		Ig-like domain-containing protein	0.0468	-0.78
A0A4X1U519		C1q domain-containing protein	0.0046	-0.80
A0A481D027		55 kDa erythrocyte membrane protein	0.0133	-0.82
A0A480XY68		Golgi reassembly-stacking protein 2 isoform 1	0.0004	-0.84
A0A4X1VGH3		Gamma-synuclein	0.0039	-0.85
A0A287AUL3	CTTN	Cortactin	0.0216	-0.86
A0A8D0Y3E5		Stearoyl-CoA 9-desaturase	0.0244	-0.90
A0A287AHI7	ADIPOQ	Adiponectin, C1Q and collagen domain containing	0.0095	-0.90
A0A286ZLW6	SAFB	Scaffold attachment factor B	0.0048	-0.91
A0A8D0N9W9		Ankyrin-1	0.0005	-0.95
A0A287AIE4	KLHL41	Kelch like family member 41	0.0333	-0.96
A0A287B883	PTMS	Parathymosin	0.0006	-0.99
A0A4X1UE92	SPTB	Spectrin beta chain	0.0022	-0.99
A0A480WYE0			0.0265	-1.01
A0A8D0UVS6		Cytochrome P450 4A11	0.0192	-1.03
A0A287BJX0	EPB42	Erythrocyte membrane protein band 4.2	0.0036	-1.07
A0A4X1T458	C3H16orf58	Chromosome 3 C16orf58 homolog	0.0003	-1.08
A0A8D0RF59	CKAP4	Cytoskeleton-associated protein 4	0.0041	-1.10
A0A4X1UAD2	AHSG	Alpha-2-HS-glycoprotein	0.0084	-1.10

A0A4X1TDP1	PTMA	Prothymosin alpha	0.0094	-1.13
A0A286ZYE7	HNRNPDL	Heterogeneous nuclear ribonucleoprotein D-like isoform a	0.0473	-1.19
A0A4X1UWI5	ETNPPL	Ethanolamine-phosphate phospho-lyase	0.0001	-1.19
A0A4X1SYX3	LOC100515788	Hemoglobin subunit beta-like	0.0184	-1.21
A0A287AI92	CA1	Carbonic anhydrase	0.0030	-1.27
A0A4X1TEJ7	BBS4	Bardet-Biedl syndrome 4	0.0380	-1.30
A0A8D1EPB8	SLC4A1	Anion exchange protein	0.0000	-1.39
A0A8D1BP89	FASN	Fatty acid synthase	0.0009	-1.56
A0A4X1SW08		IgG constant region	0.0355	-1.82
A0A4X1W9H2	RBMX2	RRM domain-containing protein	0.0002	-1.89
A0A4X1V7Z1		Ig-like domain-containing protein	0.0300	-2.23
A0A287B827	MBL2	Mannose-binding protein C	0.0321	-3.12

Table S4 The list of DEPs between the DQ and SeMet groups

Accession	Gene symbol	Protein_name	P-value	Log2FC
A0A4X1SJ49		Immunoglobulin like and fibronectin type III domain containing 1	0.0374	3.03
A0A4X1UWE1	OSTC	Oligosaccharyltransferase complex subunit	0.0126	1.94
A0A4X1SYC2	VKORC1L1	Vitamin-K-epoxide reductase	0.0025	1.86
A0A8D0TRF7	DECR1	2,4-dienoyl-CoA reductase 1	0.0353	1.36
A0A8D0MPW5	G6PD	Hexose-6-phosphate dehydrogenase/glucose 1-dehydrogenase	0.0165	1.30
A0A4X1UWI5	ETNPPL	Ethanolamine-phosphate phospho-lyase	0.0395	1.26
A0A4X1W9Y5	CD63	CD63 molecule	0.0155	1.26
A0A480NKX9		NHL repeat-containing protein 2	0.0065	1.26
A0A4X1T1L0	APOA4	Apolipoprotein A-IV	0.0095	1.16
A0A8D0IX55		60S ribosomal protein L35	0.0153	1.11
A0A4X1T6J6	RHOC	Protein phosphatase	0.0424	1.05
A0A286ZM20	SRRM1	Serine and arginine repetitive matrix 1	0.0020	1.04
A0A287B7T8	CHPT1	Cholinephosphotransferase 1	0.0235	1.03
A0A480REY2	ACTN3	Alpha-actinin-3 isoform 1	0.0276	1.00
A0A8D1BP89	FASN	Fatty acid synthase	0.0243	0.99
A0A4X1W277	RPL31	60S ribosomal protein L31	0.0151	0.99
A0A8D1D0K3		UDP-glucuronosyltransferase	0.0429	0.96
A0A8D1GB05	H6PD	Hexose-6-phosphate dehydrogenase/glucose 1-dehydrogenase	0.0039	0.95
A0A287B883	PTMS	Parathymosin	0.0120	0.94
A0A8D1BMD0		Alpha-1,4 glucan phosphorylase	0.0245	0.93
A0A8D1R2Z2		Cytochrome	0.0004	0.93
A0A287BS11	DECR2	Peroxisomal 2,4-dienoyl-CoA reductase	0.0002	0.88
A0A480VC23	PEMT	Phosphatidylethanolamine N-methyltransferase	0.0442	0.87
A0A480PQR0		Cytochrome c oxidase subunit 7A2	0.0295	0.86
A0A480TEQ4		Apolipoprotein A-II	0.0223	0.86
A0A286ZLL4	ELK4	ETS transcription factor ELK4	0.0024	0.85
A0A8D0S151		Protein kinase domain-containing protein	0.0432	0.84
A0A4X1TDP1	PTMA	Prothymosin alpha	0.0201	0.82
A0A287AD21	SUMO3	Small ubiquitin-related modifier	0.0145	0.78
A0A0B8RTV6	CCAR1	Cell division cycle and apoptosis regulator 1	0.0478	0.68
A0A4X1TXH1	COX6C	Cytochrome c oxidase subunit 6C	0.0240	0.68

A0A4X1U519		C1q domain-containing protein	0.0014	0.67
A0A4X1VKX3		Cytochrome b5 heme-binding domain-containing protein	0.0142	0.66
A0A4X1TTP1	TFRC	Transferrin receptor protein 1	0.0148	0.65
A0A0B4J2K0	FCGR2B	CD32	0.0477	0.63
A0A4X1UJS8	ATP6V0D1	V-type proton ATPase subunit	0.0300	0.63
A0A8D0NEY7		Carboxylic ester hydrolase	0.0158	0.61
A0A8D1Z724		Complement factor H-like	0.0146	0.61
A0A287BBV0	HPF1	Histone PARylation factor 1	0.0063	0.60
A0A286ZY48	EPN1	Epsin 1	0.0172	0.59
A0A4X1TDU1	COX7A1	Cytochrome c oxidase subunit 7A1	0.0421	0.59
A0A4X1T1F8		Apolipoprotein A-I	0.0117	0.59
A0A2C9F365	OSTF1	Osteoclast-stimulating factor 1	0.0001	0.59
A0A287AKG8	IRF2BP2	Interferon regulatory factor 2 binding protein 2	0.0011	-0.59
A0A0B8RW47	ADA	Adenosine deaminase	0.0009	-0.65
A0A0A7BZH1	SLA-DQB1	MHC class II antigen	0.0086	-0.66
A0A8D0WV45	CRYZ	Quinone oxidoreductase	0.0157	-0.66
A0A286ZQK4	CELF2	CUGBP Elav-like family member 2	0.0062	-0.67
A0A0B8RT27	TLR3	Toll-like receptor 3	0.0263	-0.67
A0A286K202	STEAP4	STEAP family member 4	0.0199	-0.69
A0A4X1U0E2	QPRT	Nicotinate-nucleotide pyrophosphorylase [carboxylating]	0.0075	-0.73
A0A287AEW5	LOC110259263	C-type lectin domain-containing protein	0.0094	-0.81
A0A8D0ZZA4	CAP1	Adenylyl cyclase-associated protein	0.0180	-0.85
A0A4X1V9Y6		Beta/gamma crystallin domain-containing protein 1	0.0289	-0.92
A0A287AZ54	PDXP	Pyridoxal phosphatase	0.0013	-0.96
A0A286ZRF3	SERPINB6	Serpin B6	0.0402	-1.01
A0A287A3U7	PIPOX	Sarcosine oxidase	0.0374	-1.06

Table S5 The list of DEPs between the CON and SeMet groups

Accession	Gene symbol	Protein_name	P-value	Log2FC
A0A8D0VT01	MYOM2	Myomesin 2	0.0478	3.86
A0A4X1UWE1	OSTC	Oligosaccharyltransferase complex subunit	0.0114	1.98
A0A4X1SYC2	VKORC1L1	Vitamin-K-epoxide reductase (warfarin-sensitive)	0.0016	1.74
A0A4X1VGY6		LIM domain binding 3	0.0122	1.56
A0A8D1XIV3		M20_dimer domain-containing protein	0.0292	1.53
A0A8D0PC73		Troponin C	0.0057	1.43
A0A5S6G0V8	CA3	Carbonic anhydrase	0.0382	1.42
A0A480NKX9		NHL repeat-containing protein 2	0.0043	1.38
A0A4X1TPE6		Cytochrome c oxidase subunit NDUFA4	0.0046	1.37
A0A4X1TWE4	RCN2	Reticulocalbin-2	0.0091	1.30
A0A8D0S151		Protein kinase domain-containing protein	0.0090	1.27
A0A8D0MPW5	G6PD	Hexose-6-phosphate dehydrogeanase/glucose 1-dehydrogenase	0.0104	1.26
A0A4X1W7B2	GLS2	Glutaminase	0.0316	1.16
A0A4X1V6Y4		E3 ubiquitin-protein ligase TRIM68	0.0298	1.16
A0A8D0R983		CBR4	0.0083	1.13
A0A8D1BMD0		Alpha-1,4 glucan phosphorylase	0.0163	1.04
A0A068ENU0	CYP3A22	Cytochrome P450 3A	0.0278	0.99
A0A480IXS3		Triokinase/FMN cyclase	0.0010	0.97
A0A480PQR0		Cytochrome c oxidase subunit 7A2	0.0238	0.96
A0A480ZPR2		Estradiol 17-beta-dehydrogenase 8	0.0094	0.91
A0A8D1R2Z2		Cytochrome	0.0121	0.91
A0A287BTN2	SUGCT	Succinyl-CoA:glutarate-CoA transferase	0.0178	0.91
A0A287B7T8	CHPT1	Cholinephosphotransferase 1	0.0294	0.88
A0A4X1W277	RPL31	60S ribosomal protein L31	0.0184	0.86
Q9MYT8	ATP5ME	ATP synthase subunite, mitochondrial	0.0353	0.85
A0A4X1TFL0	UGT1A6	UDP-glucuronosyltransferase	0.0148	0.83
A0A4X1U1P3	HSD17B13	17-beta-hydroxysteroid dehydrogenase 13 isoform A	0.0033	0.81
A0A287BHQ2	HMGB3	High mobility group protein B3 isoform b	0.0425	0.80
A0A286ZLB1	TRAP1	TNF receptor associated protein 1	0.0469	0.78
A0A480DNV8		UV excision repair protein RAD23	0.0178	0.77
A0A286ZM20	SRRM1	Serine and arginine repetitive matrix 1	0.0062	0.77
A0A4X1VUH1	REEP6	Receptor expression-enhancing protein	0.0015	0.76
A0A287A7E8	ENTPD5	nucleoside diphosphate phosphatase	0.0036	0.75

A0A5G2Q8I9	A2M	Alpha-2-macroglobulin	0.0426	0.75
A0A8D0I8G0	LOC100518644	Glycine N-acyltransferase-like protein	0.0013	0.75
A0A480ZAD8		Pyridine nucleotide-disulfide oxidoreductase domain-containing protein 2	0.0280	0.75
A0A481CI88		D-lactate dehydrogenase	0.0078	0.74
A0A8D0NEY7		Carboxylic ester hydrolase	0.0048	0.74
A0A287BAZ8	AASS	Aminoadipate-semialdehyde synthase	0.0334	0.73
A0A8D0I2H9	ACADL	Long-chain specific acyl-CoA dehydrogenase, mitochondrial	0.0001	0.72
A0A8D0PVV7	CAT	Catalase	0.0013	0.71
A0A480XC77		Cytosol aminopeptidase	0.0111	0.71
A0A4X1W4Y1		Cytochrome P450 family 4 subfamily A member 11	0.0286	0.71
A0A8D0NFJ4		Isovaleryl-CoA dehydrogenase, mitochondrial	0.0001	0.71
A0A1R7SPQ2	FMO3	Flavin-containing monooxygenase	0.0080	0.70
A0A4X1VKX3		Cytochrome b5 heme-binding domain-containing protein	0.0097	0.69
A0A4X1UF01		PKS_ER domain-containing protein	0.0408	0.69
A0A8D0P513		Estradiol 17-beta-dehydrogenase 2	0.0079	0.68
A0A4X1U1J6	BDH1	D-beta-hydroxybutyrate dehydrogenase, mitochondrial	0.0010	0.68
A0A4X1W9I5	METTL7B	Thiol methyltransferase 1B	0.0104	0.68
A0A8D0M6X7	GATM	Glycine amidinotransferase	0.0148	0.68
A0A287B0A0	BCO2	Carotenoid-cleaving dioxygenase	0.0222	0.67
A0A4X1W8A9	SFXN5	Sidoreflexin	0.0003	0.66
A0A480PDR7		3-ketoacyl-CoA thiolase	0.0112	0.66
A0A287ADR8	SLC27A4	long-chain-fatty-acid--CoA ligase	0.0137	0.65
A0A0B8RTV6	CCAR1	Cell division cycle and apoptosis regulator 1	0.0291	0.65
A0A8D1ELF4	PTGR1	Prostaglandin reductase 1	0.0024	0.64
A0A480VD45		Frataxin, mitochondrial	0.0038	0.64
A0A480S5V4		Phenylalanine 4-monooxygenase	0.0118	0.64
A0A5G2Q8G8	SLC2A2	Solute carrier family 2, facilitated glucose transporter member 2	0.0233	0.63
I3LL15	GNG5	Uricase	0.0077	0.63
A0A077EVP8	ATP8	ATP synthase protein 8	0.0205	0.63
A0A0B8RT34	CHDH	Choline dehydrogenase	0.0281	0.63
A0A287AC86	SLC35D1	Solute carrier family 35 member D1	0.0043	0.62
A0A4X1UUT1	RBP4	Retinol-binding protein	0.0132	0.61
A0A286ZJW9	DERL2	Derlin	0.0027	0.61
A0A480K345		Serine/threonine-protein phosphatase 6 regulatory subunit 1	0.0492	0.60

A0A287BFS1	RNPS1	RNA-binding protein with serine-rich domain 1	0.0102	0.60
A0A287A758	LOC110255172	Acyl-coenzyme A amino acid N-acyltransferase 1	0.0065	0.60
A0A4X1V7Q0		Sulfotransferase	0.0029	0.60
A0A4X1SS52	SLC27A2	long-chain-fatty-acid--CoA ligase	0.0076	0.60
A0A4X1TQJ8	HADHB	Trifunctional enzyme subunit beta	0.0413	0.59
A0A8D0Q398		Pentaxin	0.0233	-0.59
A0A0B8RW47	ADA	Adenosine deaminase	0.0137	-0.59
A0A286ZUS4	CHCHD3	Coiled-coil-helix-coiled-coil-helix domain containing 3	0.0384	-0.61
A0A287B733	FBXL2	F-box and leucine rich repeat protein 2	0.0034	-0.61
A0A4X1SSH5	MED13	Mediator of RNA polymerase II transcription subunit 13	0.0492	-0.61
A0A480F5A7		Threonine--tRNA ligase	0.0259	-0.62
A0A480TCL8		Alpha-1-acid glycoprotein	0.0076	-0.64
A0A4X1VQB8	FCER1G	High affinity immunoglobulin epsilon receptor subunit gamma	0.0110	-0.64
A0A4X1W8Z7		Alpha-1B-glycoprotein	0.0097	-0.67
A0A286ZXZ9	EPB41L2	Erythrocyte membrane protein band 4.1 like 2	0.0132	-0.67
A0A0B8RT27	TLR3	Toll-like receptor 3	0.0471	-0.71
A0A287ABA9	LOC110257570	TRPM8 channel-associated factor 2 isoform X1	0.0394	-0.72
A0A4X1ULS8	RAC2	Rac family small GTPase 2	0.0073	-0.72
A0A286ZZP8	SRP72	Signal recognition particle subunit SRP72	0.0030	-0.73
A0A4X1UXM8	CYGB	superoxide dismutase	0.0004	-0.78
A0A8D0ZZA4	CAP1	Adenylyl cyclase-associated protein	0.0082	-0.78
A0A4X1UE92	SPTB	Spectrin beta chain	0.0003	-0.79
A0A4X1UAD2	AHSG	Alpha-2-HS-glycoprotein	0.0435	-0.79
A0A480HZB3		Asparagine synthetase [glutamine-hydrolyzing]	0.0005	-0.80
A0A8D0N9W9		Ankyrin-1	0.0006	-0.81
A0A4X1TFH2		Alpha-methylacyl-CoA racemase	0.0271	-0.83
A0A287BRL8	MARCKS	Myristoylated alanine-rich C-kinase substrate	0.0477	-0.83
F1SS65	MYH4	Myosin, heavy chain 1	0.0088	-0.89
A0A287ALC1		Immunoglobulin heavy constant mu	0.0016	-0.91
A0A4X1T458	C3H16orf58	Chromosome 3 C16orf58 homolog	0.0106	-0.91
A0A8D0HYN7		Myosin light chain 1	0.0382	-0.95
A0A287BJX0	EPB42	Erythrocyte membrane protein band 4.2	0.0003	-1.00
A0A8D1EPB8	SLC4A1	Anion exchange protein	0.0000	-1.00

A0A287A6F0	MYL11	Myosin light chain 11	0.0056	-1.01
A0A8D0RF59	CKAP4	Cytoskeleton-associated protein 4	0.0212	-1.03
A0A287ANI9	RPL29	60S ribosomal protein L29	0.0480	-1.03
A0A480QMD5		IgM	0.0000	-1.04
A0A287B5V2	JCHAIN	Joining chain of multimeric IgA and IgM	0.0060	-1.09
A0A287BDA3	CYP4A23	Cytochrome P450 4A11	0.0101	-1.10
A0A287AEW5	LOC110259263	C-type lectin domain-containing protein	0.0028	-1.15
A0A4X1W9H2	RBMX2	RRM domain-containing protein	0.0092	-1.15
A0A4X1SYY0	LOC100739163	Glutathione S-transferase	0.0000	-1.24
A0A286ZRF3	SERPINB6	Serpin B6	0.0063	-1.27
A0A286ZIU5	LOC100156325	Serpin domain-containing protein	0.0153	-1.33
A0A8D0UVS6		Cytochrome P450 4A11	0.0147	-1.48
A0A4X1SW08		IgG constant region	0.0476	-1.57
