# Supplementary material for: Proteomic Analysis Reveals the Protective Effects of Selenomethionine Against Liver Oxidative Injury in Piglets
Source: Animals (Basel). 2025 Jul 7;15(13):1989. doi: 10.3390/ani15131989 (PMC12248808; doi:10.3390/ani15131989)
Supplement: Supplementary file 1 [file animals-15-01989-s001.zip › Table S1.pdf]

Table S1 Composition and nutrient levels of the basal diet

Item	Content
Corn, %	55.9
Soybean meal, %	16
Puffed soybean meal, %	11
Fermented soybean meal, %	4
Whey powder, %	6
Soybean oil, %	3
NaCl, %	0.25
Limestone, %	0.35
$CaHPO_4$, %	2
Lys-HCL, %	0.5
premix [1], %	1
Total, %	100
Nutrient level [2]	
DE, Kcal/kg	3454.37
Crude protein, %	18.52
Ca, %	0.74
P, %	0.36
Lys, %	1.26
Met, %	0.26
Thr, %	0.71
Trp, %	0.22
Se, mg/kg	0.28

[1] The premix provided the following per kg of diets: Vitamin A (trans-retinyl acetate) 2050 IU; Vitamin D3 (cholecalciferol) 220 IU; Vitamin E (dl- α-tocopherol acetate) 20 IU; Vitamin K3 0.6 mg; Vitamin B1 (thiamin) 1.8 mg; Vitamin B2 (riboflavin) 4.0 mg; Vitamin B6 (pyridoxine HCl) 2.0 mg; Vitamin B12 (cobalamin) 0.02 mg; biotin 0.09 mg; folic acid 0.45 mg; D-pantothenic acid 13 mg; nicotinic acid 20 mg; choline (as choline chloride) 550 mg; Cu (as copper sulfate) 6 mg; Fe (as ferrous sulfate) 90 mg; Mn (as manganese sulfate) 4 mg; Zn (as zinc sulfate) 90 mg; I (as potassium iodide) 0.14 mg; Se (as selenomethionine) 0.20 mg; Met (as dl-Met) 900 mg; Thr (as L-Thr) 100 mg. [2] The nutrient levels were calculated values (except for Se).
